# A twenty-year deposition record of elemental carbon in Northern Japan retrieved from archived filters

**DOI:** 10.1038/s41598-020-61067-2

**Published:** 2020-03-18

**Authors:** Naoki Kaneyasu, Kiyoshi Matsumoto, Takashi Yamaguchi, Izumi Noguchi, Naoto Murao, Teppei J. Yasunari, Fumikazu Ikemori

**Affiliations:** 1 National Institute of Advanced Industrial Science and Technology, 16-1 Onogawa, Tsukuba, 305-8569 Japan; 2 Division of Life and Environmental Sciences, University of Yamanashi, 4-4-37, Takeda, Kofu, Yamanashi, 400-8510 Japan; 3 Environmental and Geological Research Department, Hokkaido Research Organization, Kita-19 Nishi-12, Kita-ku, Sapporo 060-0819 Japan; 4 Graduate School of Engineering, Hokkaido University, Kita-13 Nishi-8, Kita-ku, Sapporo 060-8628 Japan; 5 Arctic Research Center and Global Station for Arctic Research, Hokkaido University, Kita-21 Nishi-11, Kita-ku, Sapporo 001-0021 Japan; 6 Center for Natural Hazards Research, Hokkaido University, Kita-9 Nishi-9, Kita-ku, Sapporo 060-8589 Japan; 7 Nagoya City Institute for Environmental Sciences, 5-16-8 Toyoda, Nagoya, 457-0841 Japan

**Keywords:** Atmospheric chemistry, Environmental monitoring, Environmental impact

## Abstract

The black carbon or elemental carbon (EC) content in ice and snow has been a concern in climate change studies, but time-series records have mostly been obtained from glacier ice-core samples in limited geographical locations, such as the Arctic or high mountains. This is the first study to present decade-long records of EC deposition measured at urban (Sapporo) and background (Rishiri Island) sites in Japan, in the mid-latitude zone of the eastern edge of the Asian continent. By using archived membrane filters from an acid rain study, we retrieved monthly EC deposition records of 1993–2012 in Sapporo and intermittent deposition data in Rishiri. Annual EC deposition showed large fluctuations, with a maximum in 2000–2001 and a minor increase in 2010–2011. This interannual change was moderately related to the deposition of non-sea salt SO_4_^2−^ and the collected water volume but did not reflect the estimated emission history of China. High depositions in 2000–2001 were probably caused by the transport of Asian Dust accompanied by air pollutants, which were characteristically active in these years. The findings of this study have implications for the use of observational data in validating global aerosol transport models.

## Introduction

In studies on the climatic effect of black carbon (BC; also referred to as elemental carbon (EC), light-absorbing carbon, refractory BC, or soot; hereafter denoted as it appears in the referenced literature), the importance of its effect on the broadband reflectance, i.e., albedo, of snow has been recognized since the early 1980s^1–4^. Inclusion of light-absorbing particles into snow crystals and/or their direct deposition onto the snow surface reduces the snow albedo and ultimately affects the aging process of snow grains and accelerates snow melt^5^.

Currently available data used to validate numerical models for estimating snow-albedo reduction have limitations due to the locality and duration of sample collection. Since the pioneering work in the early 1980s^6^, BC in the snowpack has generally been measured in the Arctic region^7^, whereas measurements in mid-latitude regions have been relatively limited. In addition, much of the concentration data in the snowpack has been reported based on single-shot or one-winter-long samplings, and time-series measurements over multiple years are limited to a small number of studies^8–12^, with the longest sampling campaign extending over three and a half years^13^.

Another type of time-series data has been obtained as historical records from ice-core samples drilled from glaciers in the Himalayan mountain range^14–16^, Tibetan Plateau^17–19^, Eastern Pamirs^20^, European Alps^21–24^, and Greenland^25^. Ice-core records can reveal the concentrations of BC in ice layers up to several centuries ago, which enables the estimation of past emissions from biomass burning and fossil fuel use^17,23–25^. In particular, time-series records from Asia are expected to contain the history of emissions from China, where current BC emissions are estimated to be the highest^26–28^. However, glaciers on the Asian continent are located west of the industrialised areas in China and are, therefore, located upwind of the prevailing westerlies.

To validate the models that reproduce global BC deposition for the purpose of estimating snow-albedo reduction, BC deposition data collected over longer time periods at mid-latitudes, where emissions are concentrated, would be valuable. We addressed this issue by using a by-product of another research project, namely an acid deposition study, in an urban and a background site in northern Japan. In the 1980s, with increasing public concern about acid deposition, environmental research institutions in Japan began collecting acid deposition samples by filtering bulk-deposition samplers^29^, which did not discriminate between wet and dry depositions. A membrane filter was installed inside the sampler to remove insoluble particles, mainly soil dust, from precipitation collected by an upward-facing funnel. Although these membrane filters were normally discarded after sampling, an environmental research institution in Hokkaido Prefecture, Japan, has preserved these used membrane filters since the 1990s.

**Figure 1:**
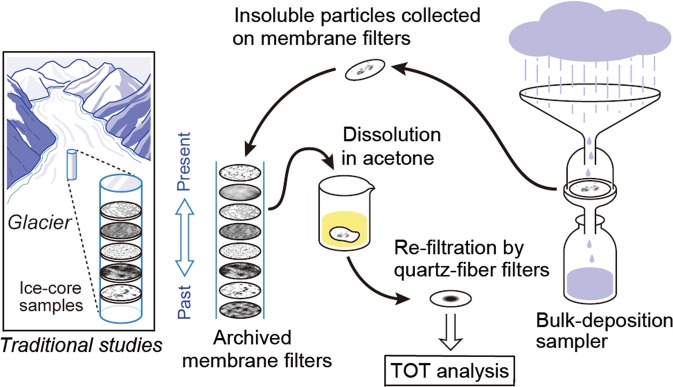
Schematic showing the concept of this study. TOT denotes the thermal-optical transmission used in particulate carbon analysis.

If the captured particles on the archived membrane filters, which span an extended period of sampling, could be analysed, they would be equivalent to ice-core samples containing deposited particles in layers (Fig. 1). We subsequently devised a technique to analyse these particles and retrieved the deposition record of EC (an alternative measure to BC), collected over 20 years at an urban site (Sapporo) in northern Japan. Additionally, an intermittent deposition record was retrieved from samples collected at a background site (Rishiri Island). An advantage of our method is that dating with one-month resolution has already been achieved. This enabled us to reproduce seasonal variations in deposition, which most ice-core studies were unable to achieve. This study presents the first long-term EC deposition record on the eastern edge of the Asian continent. The results have implications for the use of observational data in validating global aerosol transport models.

## Methods

### Sampling apparatus and sites

In 1983, the Environment Agency of Japan (currently the Ministry of Environment) conducted the first nationwide study (Phase 1) of the Japanese Acid Deposition Survey (JADS) program. In JADS Phase 1, precipitation was collected by filtering bulk-deposition samplers. Schematics and a description of the samplers used are provided in Supplementary Fig. S1. Chemical analyses of the water samples collected in the JADS are described elsewhere^30^. The membrane filters installed in the samplers were retrieved once a month at the same time the water samples were collected, with some minor exceptions due to weekly sampling conducted for several intensive studies. When the filter was replaced at the end of each month, the funnel of the sampler was rinsed with distilled-deionised water (200 mL). The Hokkaido Institute of Environmental Sciences (currently the Hokkaido Research Organization) has been operating these samplers since 1983 and has preserved some of the membrane filters since 1993.

**Figure 2:**
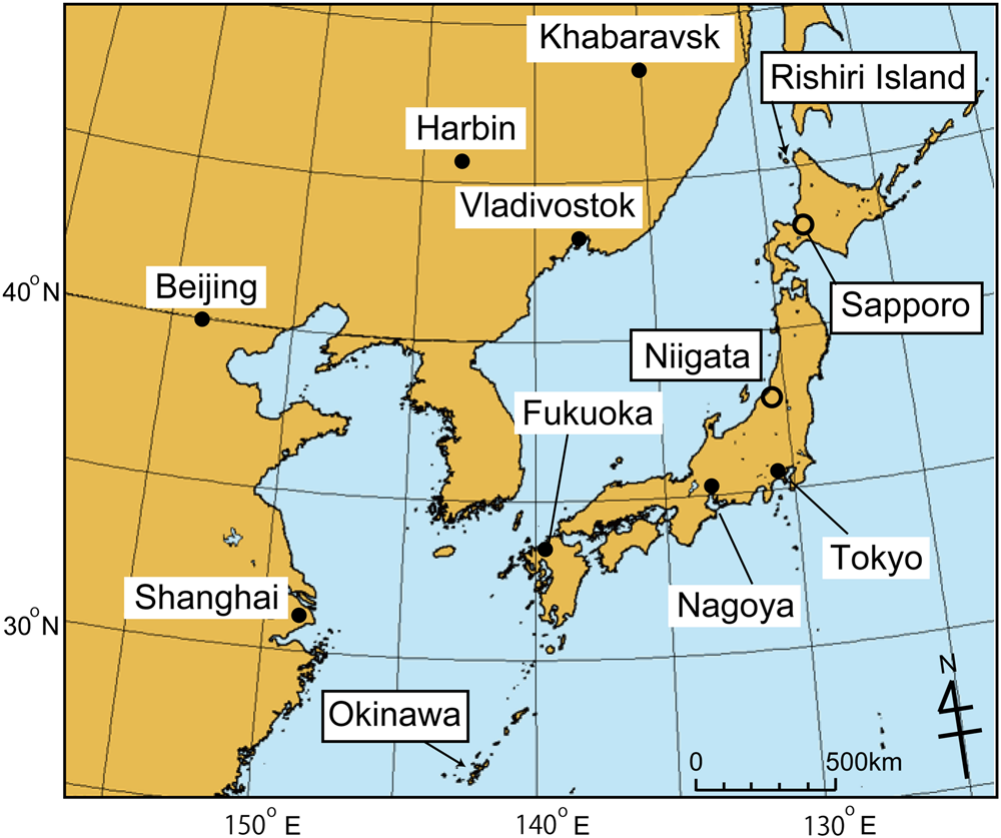
Map of the study region in East Asia. Location names corresponding to data mentioned in the text are enclosed in the solid rectangular frames.

One sampler was operated on the rooftop (12 m above ground level) of a building at the Hokkaido Institute of Environmental Sciences (43 °04′54″N, 141 °20′00″E, 15 m above sea level (asl.)). The sampling location was in Sapporo, Japan, the capital city and commercial centre of Hokkaido Prefecture (Fig. 2). Another sampler was deployed at the Rishiri National Acid Deposition Monitoring Station (45 °07′11″N, 141 °12′26″E, 50 m asl.) on southwestern Rishiri Island, a background site in northernmost Hokkaido (Fig. 2). Sampling on Rishiri Island was interrupted from 2001 to 2007, and sampling was not conducted during the heavy snow season (January–March) in several years.

### Extraction and analytical procedures

The membrane filters used by the Hokkaido Institute of Environmental Science were mixed cellulose ester filters (pore size: 0.8 μm, 47 mm ϕ; ADVANTEC A080A047A, Advantec Toyo Ltd., Japan), which are acetone soluble. First, to remove carbonate from soil dust particles, the membrane filters loaded with insoluble particles were soaked in 10 mL of aqueous HCl (2 N) in a glass beaker and heated on an electric heater. During heating, the HCl solution became an azeotropic mixture with water. The heater was turned off before the solution completely evaporated and the filter in the beaker was left at 20–25 °C until it was dry. After the removal of carbonate, 25 mL of acetone was added to the beaker, which was then placed in an ultrasonic bath for 5 minutes to dissolve the remaining filter material. The solution was then filtered through a nylon net (mesh opening, 5 μm; NYTAL, Sefar AG, Switzerland) to remove coarse soil particles, and 5 mL of acetone was poured onto the remaining soil particles on the net to wash out the particulate carbon. Finally, the extraction was filtered through two-ply quartz-fibre filters (25 mm ϕ; Whatman QMA, GE Healthcare life Sciences, Pittsburgh, PA) mounted on a glass filtration funnel. The quartz-fibre filters were pre-treated by heating in air at 850 °C for 30 minutes using an electric furnace to remove any carbonaceous material from the blanks.

The sample-loaded quartz-fibre filters were analysed using a thermo-optical transmittance (TOT) carbon analyser (Sunset Laboratory, Tigard, OR) according to the Interagency Monitoring for Protected Visual Environments (IMPROVE) protocol^31^, which separates EC from organic carbon in a helium stream with a stepwise heating process. The instrument was calibrated with sucrose solution standards. Repeated analyses of a PM_2.5_ filter sample collected in an urban area (Fukuoka, western Japan) yielded a coefficient of variation of less than 15% (*n* = 19) for EC with this instrument. In deposition studies of carbonaceous particles, a thermal-oxidation analysis method^32^ has often been employed. Our analytical results were compared with this method (Supplementary Information).

In previous studies, the uncertainty in capture efficiency was discussed when quartz-fibre filters were used to separate particles suspended in water^33–36^ because this type of filter was originally produced for the filtration of particles suspended in air. In the present study, we used two quartz-fibre filters placed in series for filtration and corrected the EC mass captured on the first filter. The correction procedure and discussion on the transmission efficiency of carbonaceous particles through the filters are described in the Supplementary Information.

## Results and Discussion

### Interannual variation in deposition flux

**Figure 3:**
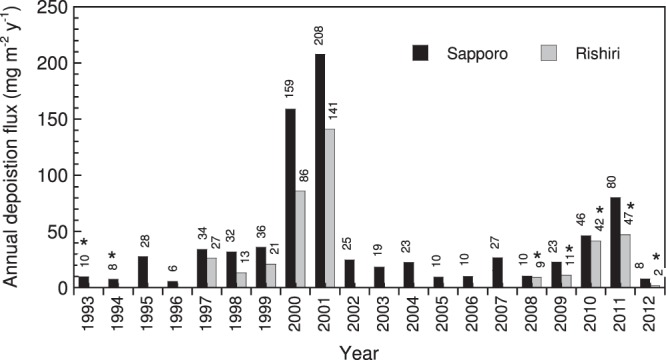
Annual bulk deposition of elemental carbon (TOT-IMPROVE) in Sapporo and Rishiri from 1993 to 2012. Bars marked with asterisks represent incomplete annual data because of missing samples from January–March. Numbers above the bars denote deposition flux values.

Annual deposition (mg m^−2^ y^−1^) in Sapporo was calculated for 1993–2012 by summing the monthly deposition in each year (Fig. 3). Samples were not collected during January–March 1993 and 1994, making annual deposition data incomplete for these years. Therefore, we discuss only the interannual changes based on data from 1995 to 2012. Some of the annual deposition data for Rishiri are also missing, similar to Sapporo, since sampling was not performed when the site was inaccessible because of deep snow (January–March).

As seen in Fig. 3, an increasing or decreasing trend over the sampling period was not observed in Sapporo. Instead, an extremely high deposition in 2000 and 2001 was prominent, and a second noticeable increase in deposition occurred in 2011. For example, the annual deposition in 2000 and 2001 in Sapporo were 3.7 and 4.8 times higher than the 1995–2012 mean, respectively. The locations of the sampling sites on the eastern edge of the Asian continent were expected to be suitable for detecting the emission history of carbonaceous particles from China, associated with its economic growth and the implementation of pollution reduction measures. However, the deposition record obtained does not reflect the BC emission history of China that has been estimated in two independent studies. An emission estimate during 1996–2010 shows a minimum in 2000 with progressive increase until 2008^37^. Another estimate shows a sharp local maximum in 1996 prior to a local minimum in 2000, with a plateau in the late 1990s^38^. The local minimum in 2000 estimated for emissions in China coincided with an extremely high EC deposition observed in Sapporo. The continued increases in emissions in China during the early 2000s were not observed in the deposition record from Sapporo.

During the spring–early summer of 2003, a massive boreal forest fire occurred in Eastern Siberia that transported thick plumes of carbonaceous particles at high altitudes to Japan^39^ and likely affected the surface aerosol concentrations in Nagoya, central Japan^40^, and Rishiri^41^. In Sapporo, surface air quality was possibly affected by the Siberian forest fires in 2003 and 2008^42^. However, such prominent events did not increase EC deposition in Sapporo. This suggests that even if high concentrations of carbonaceous particles were transported to the study area, they would not necessarily have increased the deposition flux. Previous studies^12,43^ have noted that the occurrence of precipitation coincident with the arrival of carbonaceous particles is likely required to cause high levels of deposition. The possible concurrence of precipitation and high concentration of air pollutants in the atmosphere will be discussed in the last section as a cause of increased disposition in 2000 and 2001.

**Figure 4:**
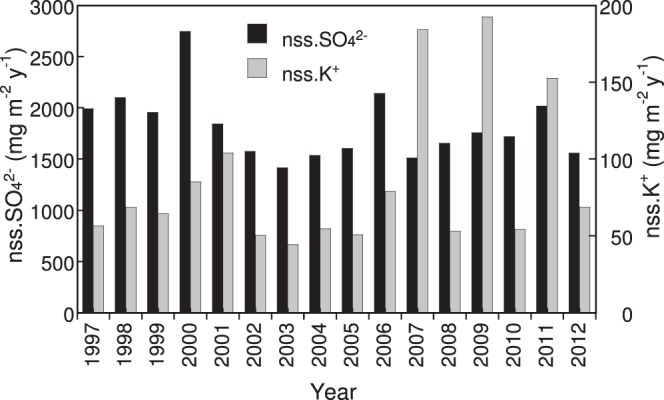
Annual bulk deposition of non–sea salt (nss.) SO_4_^2−^ and nss.K^+^ from 1997 to 2012 in Sapporo measured according to the protocol of the Acid Deposition Survey Phase 1 program.

Potential emission sources of carbonaceous particles, such as fossil fuel combustion, have been indicated by comparison with other chemical components in melted water from ice-core samples^17^ or in precipitation^44^. For comparison with our retrieved EC deposition data, the concentrations of inorganic ions, mass of water-insoluble material, and volume of collected water in the bottle were available from 1997 onward and were measured according to the JADS program protocol. The deposition fluxes of ionic components were calculated by multiplying the concentrations and volume of collected water. In Sapporo, the time series of annual non-sea-salt (nss.) SO_4_^2−^ and nss.K^+^ deposition (Fig. 4) did not fluctuate greatly, as was the case with EC. The non-sea salt correction is described in the Supplementary Information.

**Figure 5:**
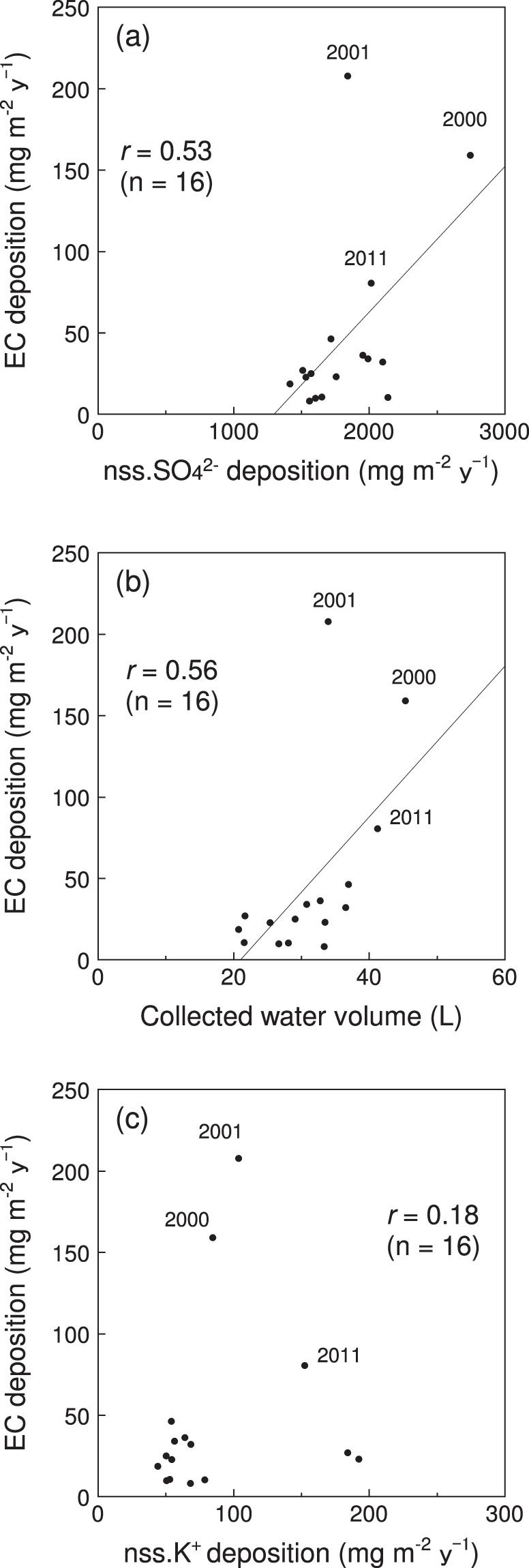
Relationship of annual elemental carbon (EC) deposition in Sapporo with (**a**) non–sea salt (nss.) SO_4_^2−^ deposition, (**b**) the volume of water collected by the sampler, and (**c**) nss.K^+^ deposition.

The annual deposition flux of EC in Sapporo moderately correlated with that of nss.SO_4_^2−^ (Pearson correlation coefficient *r* = 0.53, significance level *p* < 0.05) for 1997–2012 (Fig. 5a). The collected water volume moderately correlated with the annual EC deposition (*r* = 0.56, *p* < 0.05) for the same period (Fig. 5b). On the other hand, the correlation of annual EC deposition with that of nss.K^+^ was weak, and these parameters were considered to be statistically unrelated (*r* = 0.18, *p* < 0.05). These comparisons indicate that the annual EC deposition in Sapporo was, at least to a certain extent, associated with pollutants from sulphur-containing combustion sources, such as coal combustion, and affected by the amount of precipitation. Although the effect of biomass-burning emissions was hardly identifiable from the correlation coefficient with nss.K^+^, several regression lines in the scatterplots may be drawn between EC and nss.K^+^ deposition (Fig. 5c).

As the analytical procedure adopted in our study was relatively complicated and has not been used in previous studies, the validity of the measured values should be assessed. In 2010, the deposition flux of TOT-EC was measured in a forested area in Niigata, Japan, with the use of filtering bulk-deposition samplers^45^. The sampling apparatus and analytical instrumentation were similar to those in our study, with the only difference being that the quartz-fibre filters were directly installed in bulk-deposition samplers to collect carbonaceous particles. The annual deposition of TOT-EC observed outside the forest canopy was 71 mg m^−2^ y^−1^. In Cape Hedo of Okinawa, Japan, the refractory BC in rainwater was measured using a single-particle soot photometer, and the resultant annual deposition flux was 66 mg m^−2^ for the period of Apr. 2010–Mar. 2013^12^. Compared to these reported fluxes measured in Japan during the same period, the retrieved annual deposition flux in Sapporo in 2010 (46.2 mg m^−2^ y^−1^) and during Apr. 2010–Mar. 2013 (39.5 mg m^−2^ y^−1^) was within the same order of magnitude. The 1995–2012 averaged annual EC deposition in Sapporo (43.4 mg m^−2^ y^−1^) also agrees with the BC deposition of 44 mg m^−2^ y^−1^ calculated in a global transport model for the 30–60 °N latitude zone^46^.

Notably, the annual deposition at the background Rishiri site was not substantially smaller than that in Sapporo (Fig. 3). The Rishiri/Sapporo annual deposition ratio in 1997–2001, during which complete annual data were available for both sites, ranged between 0.42 and 0.78. This suggests that even in a populated metropolitan area, such as Sapporo, the annual deposition of EC is dominated by regional deposition rather than the immediate deposition of urban air pollution emitted *in situ*.

### EC concentrations in collected water

**Figure 6:**
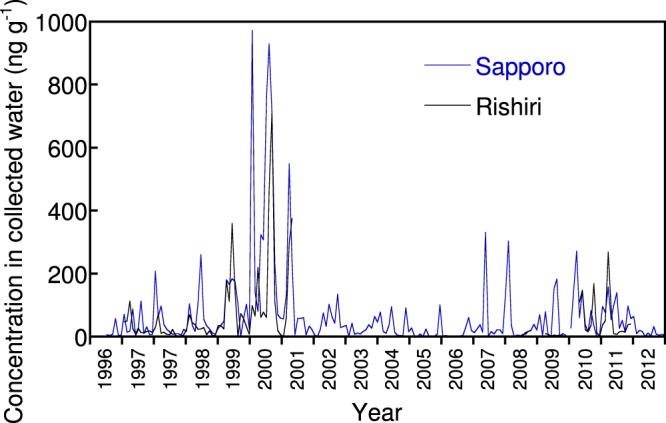
Monthly mean concentrations of elemental carbon (TOT-IMPROVE) in the collected water in Sapporo and Rishiri from 1996 to 2012.

In previous studies, BC or EC measurements in the surface snowpack or ice-core samples have mostly been presented as concentrations in melted water and not as deposition fluxes. For comparison purposes, the measured EC values from Sapporo and Rishiri were converted to concentrations in precipitation by dividing the mass by the volume of collected water. The calculated monthly mean EC concentrations in collected water in Sapporo and Rishiri for the period of 1997–2012 are shown in Fig. 6. The mean concentration in Sapporo during 1997–2012 was 70.6 ng g^−1^ (*n* = 191), whereas that of Rishiri was 56.4 ng g^−1^ (*n* = 101). In this expression, the amount of EC was also noticeably high in 2000 and 2001. For example, in Sapporo, the concentration ratios of 2000 to 1997–2012 and 2001 to 1997–2012 were 2.6 and 5.2, respectively, which were similar to the enhancement ratios of the deposition in these years. This suggests that an increased deposition in these years occurred in the form of wet rather than dry deposition.

The concentrations in Sapporo and Rishiri were higher than those of high mountainous sites in North America^6,47^ and European background sites^10,48^, probably reflecting the relative proximity to the intense emission areas on the Asian continent. Recent extensive surveys in Northeast China have reported much higher BC concentrations (measured with an integrating sandwich method) in the surface snowpack^49,50^. Among the reported measurements from the mid-latitudes (Supplementary Table S1), the time-series data from the Sapporo snowpack samples collected during the winters of 2007–2008 and 2008–2009^11,51^ overlap with the collection periods of our data. They show values of 0.01–0.8 and 0.005–10 ng g^−1^ EC (thermo-optical reflectance) for the respective winters (visual read-out from the figure by Aoki *et al*.^11^). The retrieved EC concentrations from the archived filters for the corresponding periods were 0.7–1.5 and 3.1–6.9 ng g^−1^, respectively. Although the lowest concentrations from the archived filters were several orders of magnitude greater than the corresponding snowpack data, the highest concentrations were within a factor of two of the snowpack results.

### The possible cause of high deposition in 2000 and 2001

**Figure 7:**
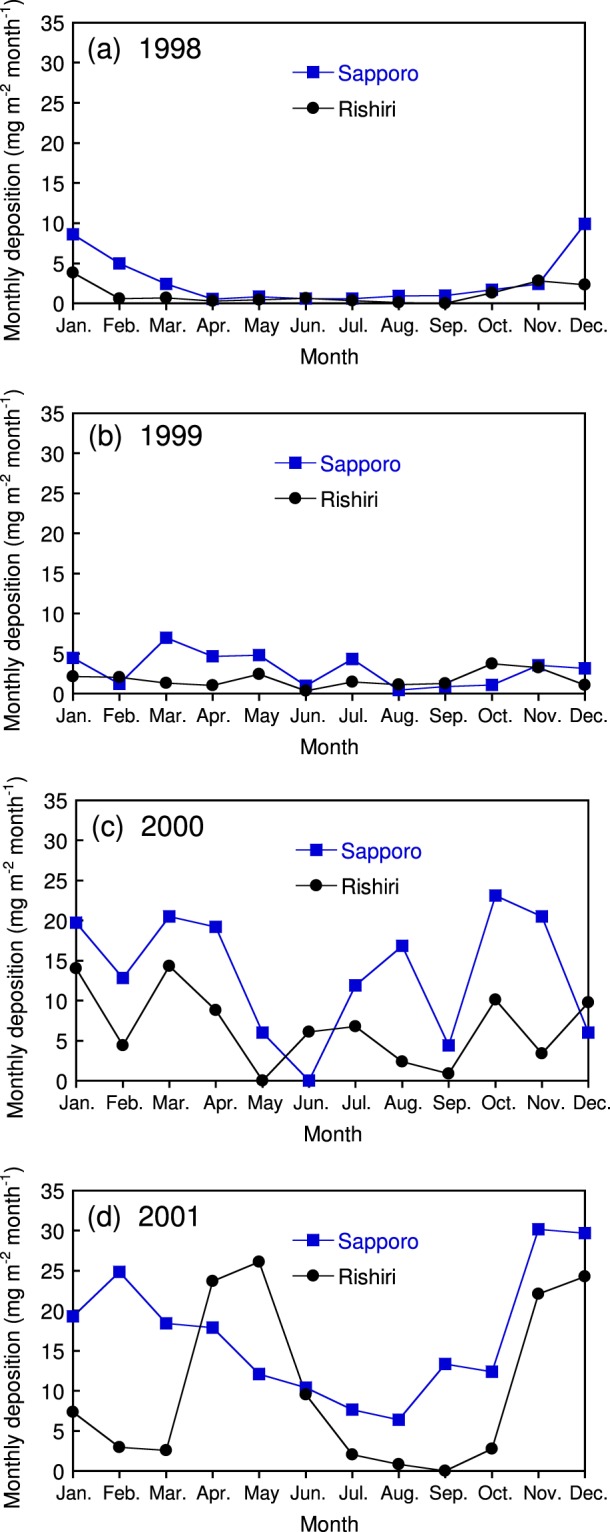
Seasonal variations in monthly elemental carbon deposition in Sapporo and Rishiri measured in (**a**) 1998, (**b**) 1999, (**c**) 2000, and (**d**) 2001.

As discussed above, the high deposition in 2000 and 2001 cannot be explained by the correlation with the annual deposition of nss.SO_4_^2−^, nss.K^+^, and the collected water volume. These high values were not caused by a contamination in the extraction process because the colour of the membrane filters collected from the filtering bulk-deposition samplers was already dark (Supplementary Figs. S3 and S4). For a more detailed observation, seasonal variations in the monthly EC deposition in Sapporo and Rishiri during 1998–2001 are presented in Fig. 7a–d. The seasonal variation patterns differed greatly between years, regardless of high deposition years (2000 and 2001) or low deposition years (1998 and 1999). Although the monthly deposition was generally greater in Sapporo than in Rishiri, a higher deposition in Rishiri was observed in April–May 2001. These spring months are known as the dominant outflow season for Asian Dust and air pollutants to the North Pacific Ocean^52,53^. A spring maximum in deposition was also reported for refractory BC collected at Cape Hedo in Okinawa, a southern background site in Japan, and was attributed to the coincidence of higher precipitation amounts and higher BC concentrations in the Asian air masses transported frequently in this season^12^.

**Figure 8:**
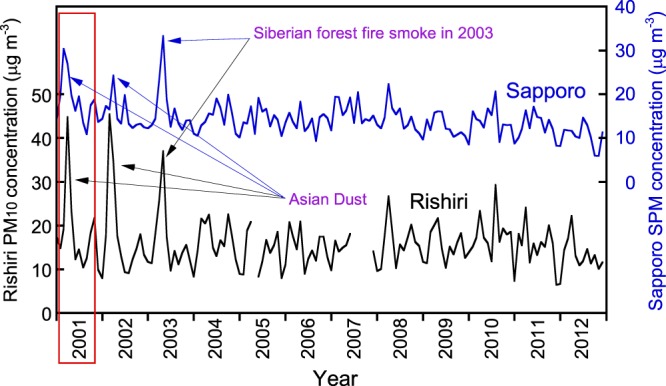
Monthly mean concentrations of suspended particulate matter (SPM) in Sapporo and PM_10_ in Rishiri from 2001 to 2012.

Therefore, to find the cause of high deposition in a particular season, it is necessary to compare the temporal change in the concentration of EC in the atmosphere and the deposition flux. However, we do not have atmospheric concentrations of EC in Sapporo and Rishiri that date back to the early 1990s. As an alternative measure, monthly mean concentrations of suspended particulate matter (SPM, roughly equivalent to PM_4.5_ to PM_6_) in Sapporo and PM_10_ in Rishiri from 2001 to 2012 are available (Fig. 8). SPM is a regulatory measure of particulate air pollutants in the ambient quality standards in Japan, and has been monitored in urban areas since the 1970s. Measurements of PM_10_, simultaneously with PM_2.5_, have started in the beginning of 2001 in a limited number of air monitoring stations as a trial investigation. In both SPM and PM_10_ records, extremely large increases in the concentrations were observed in 2001, 2002, and 2003. Among them, the concentration peak in 2003 can be attributed to the advection of smoke from Siberian forest fires as mentioned earlier, and those in 2001 and 2002 are regarded as Asian Dust in view of the ratio of PM_2.5_ and PM_10_. In the East Asian countries, the transport of Asian Dust was active in the years of 2000, 2001, and 2002, and was discussed in connection with the increased dust storm days in North-Northeastern China^54,55^.

**Figure 9:**
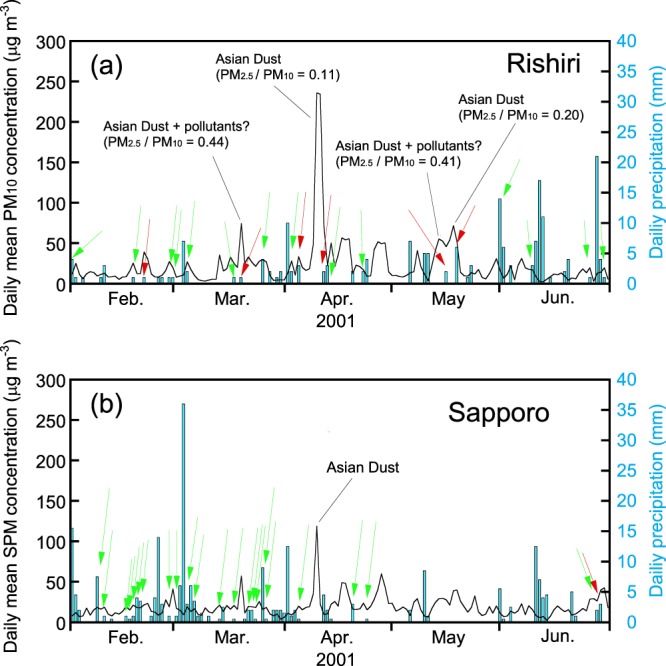
(**a**) Daily precipitation and daily mean concentration of PM_10_ from February to June 2001, in Rishiri. (**b**) Daily precipitation and daily mean concentration of SPM from February to June, 2001, in Sapporo. Green arrows indicate the concurrence of the daily mean concentration of particulate matter >15 μg m^−3^ and daily precipitation >0.5 mm, and red arrows indicate the concurrence of the daily mean concentration of particulate matter >30 μg m^−3^ and daily precipitation >0.5 mm.

From Fig. 8, it is apparent that the seasonal variation patterns of PM_10_ and SPM in 2001 surrounded by the red rectangle are similar to those of the EC deposition shown in Fig. 7(d). In this period, the EC deposition in Rishiri was active in April, May, and June, while that in Sapporo showed a maximum in February, followed by a gradual decrease until summer. As a case study, we superimposed the daily precipitation values on the daily mean concentrations of the particulate matter in Rishiri and Sapporo for the period from February to June 2001 (Fig. 9), during which the deposition patterns of EC differed greatly between the sites. The concurrence of precipitation and relatively high concentrations (>15 μg m^−3^) of particulate matter days was dispersed in Rishiri, whereas that in Sapporo was concentrated in the first half of the period. In Rishiri, a concurrence of precipitation and high concentrations of PM_10_ (>30 μg m^−3^, marked with red arrows in the figure), presumably the Asian Dust or a mixture of pollutants and Asian Dust, were observed several times. A rough estimate of the aerosol size distribution is available in Rishiri as the ratio of PM_2.5_/PM_10_ for a limited period. For example, this ratio was 0.11 for the extremely high aerosol concentration on April 10 (PM_10_: 253 μg m^−3^), which is a relatively small value. On the other hand, there were other high aerosol concentration days with larger PM_2.5_/PM_10_ ratios, such as March 20 (PM_10_: 74 μg m^−3^, PM_2.5_/PM_10_ = 0.44) and May 15 (PM_10_: 53 μg m^−3^, PM_2.5_/PM_10_ = 0.41). These days of high PM_10_ concentrations with relatively high PM_2.5_/PM_10_ ratios indicate the arrival of Asian Dust accompanied by pollution-type aerosols predominantly in the PM_2.5_ range. Asian Dust mixed internally or externally with pollution-type aerosols has received increasing attention in view of its radiative effect^56,57^. The wet deposition of such polluted Asian Dust or pollution-type aerosols in its wake is the probable cause of the increased EC deposition in April and May in Rishiri. On the other hand, the wet deposition of EC in Sapporo was concentrated in February and March, which coincided with frequent precipitation events. In summary, the pronounced high EC deposition in 2000 and 2001 is attributable to the wet deposition of Asian Dust, characteristically active in these years, and the simultaneous transport of pollution-type aerosols. An open question in this interpretation might be related to why the deposition of nss.SO_4_^2−^ was not notable in these years as compared to that of EC. A probable cause for this observation might be that while initially emitted EC with a hydrophobic nature gradually acquires hydrophilicity with transport time, nss.SO_4_^2−^ is totally water-soluble from the beginning of the transport. Thereby, nss.SO_4_^2−^ is preferentially removed from the atmosphere during the whole long-range transport via acting as cloud condensation nuclei and subsequent wet-deposition, while EC transported simultaneously can survive in the atmosphere and wet-deposit relatively far from the emission sources.

Based on comparisons with three other independent measurements, i.e., the deposition fluxes in Niigata and Cape Hedo, and the concentrations in the Sapporo snowpack, we believe that our measurements are reliable. The novel finding of this study is that EC deposition had a large interannual variation at the endpoint of the long-range transport, which exceeded the variations in other source marker substances, such as nss.SO_4_^2−^. In studies evaluating the radiative forcing induced by BC in surface snow and ice, global transport models have been used to estimate BC and/or other light-absorbing aerosol concentrations in precipitation or the surface snowpack^58–60^. Deposition fluxes derived from these models are, as a regular procedure, compared and validated by observations available at the time. In view of our results, validating or tuning models using observational data from single-shot or one-season sampling may lead to erroneous results.

## Supplementary information

Supplementary Information.

## Data Availability

The data used in this study, except those included in this published article and its Supplementary Information files, are available by contacting the corresponding author on reasonable request.
