# Supplementary material for: A twenty-year deposition record of elemental carbon in Northern Japan retrieved from archived filters
Source: Sci Rep. 2020 Mar 18;10:4520. doi: 10.1038/s41598-020-61067-2 (PMC7080725; doi:10.1038/s41598-020-61067-2)
Supplement: Supplementary file 1 — Supplementary Information. [file 41598_2020_61067_MOESM1_ESM.docx]

**Supplementary Information**

A twenty-year deposition record of elemental carbon
in Northern Japan retrieved from archived filters

Naoki Kaneyasu*^1^, Kiyoshi Matsumoto^2^, Takashi Yamaguchi^3^, Izumi Noguchi^3^, Naoto Murao^4^, Teppei J. Yasunari^5, 6^ & Fumikazu Ikemori^7^

^1^ National Institute of Advanced Industrial Science and Technology, 16-1 Onogawa, Tsukuba 305-8569, Japan

^2^ Division of Life and Environmental Sciences, University of Yamanashi, 4-4-37, Takeda, Kofu, Yamanashi 400-8510, Japan

^3^ Environmental and Geological Research Department, Hokkaido Research Organization, Kita-19 Nishi-12, Kita-ku, Sapporo 060-0819, Japan

^4^ Graduate School of Engineering, Hokkaido University, Kita-13 Nishi-8, Kita-ku,
Sapporo 060-8628, Japan

^5^ Arctic Research Center and Global Station for Arctic Research, Hokkaido University, Kita-21 Nishi-11, Kita-ku, Sapporo 001-0021, Japan

^6^ Center for Natural Hazards Research, Hokkaido University, Kita-9 Nishi-9, Kita-ku,
Sapporo 060-8589, Japan

^7^ Nagoya City Institute for Environmental Sciences, 5-16-8 Toyoda, Nagoya 457-0841, Japan

*Corresponding Author, E-mail: kane.n@aist.go.jp

**Table of Contents**

Description of the filtering bulk-deposition sampler (Fig. S1) Page S3

Comparison with thermal oxidation method of Cachier et al. (1989) (Fig. S2) Page S4

Correction of particle capture efficiency by filters Page S6

Non-sea-salt correction Page S8

Table S1 Page S9

Example of membrane filters loaded with deposited particles (Figs. S3 and S4) Page S11

References Page S13

**Description of the filtering bulk-deposition sampler**

The sampler consists of a PTFE-coated funnel (inner diameter, 380 mm) and PTFE tubing that carries the collected precipitation (rain or melted snow) through the bottom of the funnel to a polyethylene bottle (20 L) that stores the water (Fig. S1). The funnel is heated slightly to melt snow or graupel when the ambient temperature is below 0 °C. Halfway down the tube and above the water storage bottle, a polysulfone filter holder is installed to mount a membrane filter that removes insoluble materials from the collected water.

**
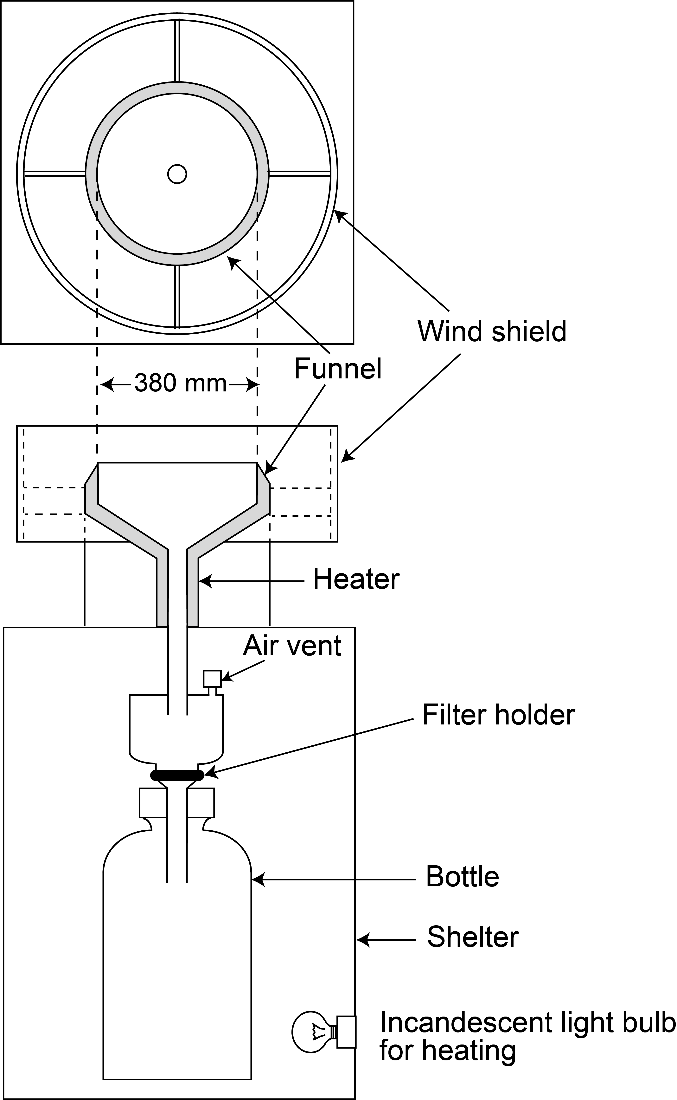
**

**Figure S1.** Schematic of the filtering bulk-deposition sampler system.

**Comparison with thermal oxidation method of Cachier et al. (1989)**

For comparison, some filters with high loads were divided into several sections. Aliquots were treated using a thermal-oxidation technique to separate EC from organic carbon in a pure oxygen (99.999%) stream at 340 °C for 2 hours (Cachier et al. 1989) and were then analysed using flame-ionization detection gas chromatography after combustion in a CN-analyser (Kaneyasu et al. 1995). As shown in Fig. S2, thermo-optical transmittance EC analyses (Interagency Monitoring for Protected Visual Environments protocol) by means of a Sunset Laboratory analyser and the Cachier et al. (1989) method show a linear relationship with a coefficient of determination *R*^2^ of 0.83 (*n* = 41). This enabled us to compare our results with those of other studies and interpret the results accordingly.


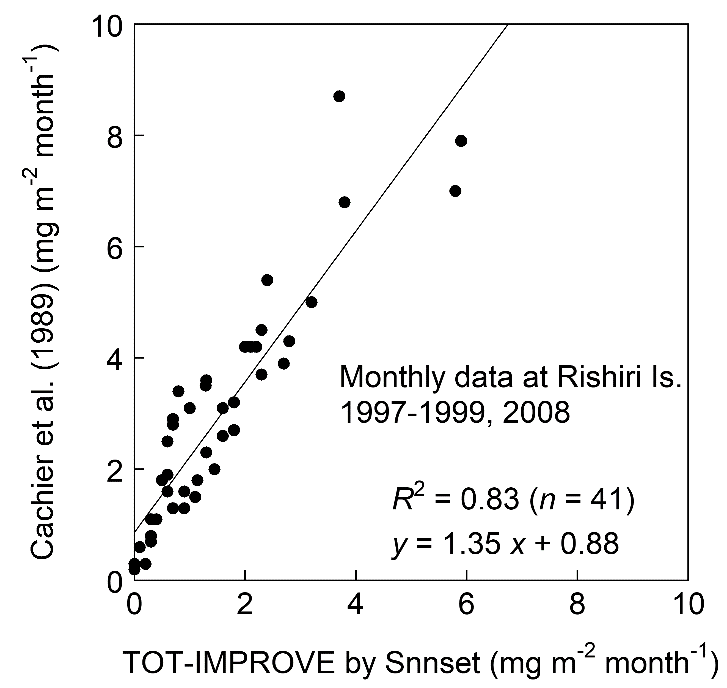


**Figure S2.** Comparison of elemental carbon (EC) concentrations determined using a thermal-oxidation method by Cachier et al. (1989) and a thermo-optical transmittance (TOT) method with a Sunset Laboratory analyser under the Interagency Monitoring for Protected Visual Environments (IMPROVE) protocol.

The reason for adopting the Cachier et al. (1989) method for comparison was that many studies on precipitation, snowpack, and ice cores (Ducret and Cachier 1992; Lavanchy et al. 1999; Jenk et al. 2006; Ming et al. 2008; Liu et al. 2008; Xu et al. 2009; Thevenon et al. 2009) have adopted this or similar analytical methods. Chylek et al. (1987) reported concentrations in surface snow at low-latitude sites (New Mexico and Texas, USA), and the separation procedure for EC from organic carbon (using air rather than inert gas and a separation temperature of 350–400 °C) was similar to that of Cachier et al. (1989).

**Correction of particle capture efficiency by filters**

For a particle suspended in a liquid, capture efficiency by a quartz-fibre filter may vary according to the physical properties of the suspended particle. In this study, the penetration rate of particles through the quartz-fibre filter *P*_quartz_ was defined as follows:

*P*_quartz_ = *C*_2_/*C*_1_ (Eq. S1)

This equation was used to calculate the capture efficiency 1 – *P*_quartz_ of the deposited EC particles by the filter, where *C_1_* and *C_2_* are the determined EC on the first and second filters, respectively, laid in series. Then, the corrected EC concentration *C* could be obtained:

*C* = *C*_1_/(1 – *P*_quartz_) (Eq. S2)

This correction assumes that *P*_quartz_ does not change according to the amount of insoluble materials retained on the filters. In most deposition samples, *P*_quartz_ was less than 0.01 (capture efficiency > 99%), i.e., the determined EC amount on the second filter was less than or close to the detection limit, with some exceptions of several extremely high values of *P*_quartz_ obtained from the summer samples from Rishiri Island, such as 0.65 (capture efficiency = 35%, July 2009) and 0.43 (capture efficiency = 57%, September 2008). Notably, for 2001, two-ply filtration was not conducted for deposition samples in either Sapporo or Rishiri. This was the first round of analysis in this study, and we decided to subsequently evaluate the capture efficiency of the quartz-fibre filters. Thus, the measured deposition in both Sapporo and Rishiri in 2001 may be underestimated because the capture efficiency correction was not applied.

In previous studies, capture efficiencies of BC by single quartz-fibre filters were reported to be 50–80% (Ogren et al. 1983) and 30% when laboratory standards of pure water and BC were tested (Hadley et al. 2010). The capture efficiencies of two of the three samples were close to 100% (undetectable amounts of EC were measured on the second filter), one was approximately 70% (Aamaas et al. 2011), and some were less than 38% (Torres et al. 2014). The capture efficiency in our study was generally far higher than the aforementioned results. Although the mechanism is unclear, dissolving the mixed cellulose ester membrane filter in acetone may have had a “salt-adding” effect that increased the capture efficiency of quartz-fibre filters, as noted by Torres et al. (2014).

For the membrane filters installed in the filtering bulk-deposition sampler, the penetration rate of particles in the liquid phase *P*_membrane_ had to be examined in the same manner. We used a reference urban aerosol sample (CRM No. 28, National Institute of Environmental Study, Japan) and found that *P*_membrane_ was below 0.01 in all filtration tests (*n* = 5).

**Non**-**sea**-**salt correction**

To eliminate the contribution of ionic species contained in sea-salt particles to the measured concentrations in precipitation, non-sea-salt (nss.) fractions were calculated for each species via measured Na^+^ concentrations and known sea salt composition (Seinfeld and Pandis, 1998):

nss.SO_4_^2-^ = SO_4_^2-^ – 0.25 × Na^+^ (Eq. S3)

nss.K^+^ = K^+^ – 0.036 × Na^+^ (Eq. S4)

nss.Ca^2+^ = Ca^2+^ – 0.038 × Na^+^ (Eq. S5)

**Table S1.** Concentrations of elemental carbon (EC) and black carbon (BC) measured in precipitation, melted snow, and melted snowpack collected at mid-latitudes.

| Location; sampling duration | Sample type; mean concentration (range) | Analytical method | Reference |
| --- | --- | --- | --- |
| Cascade mountains, WA, USA (914 m asl.); spring 1980 | Snowpack; 22–59 ng g^-1^ | Light absorption (BC) | Grenfel et al. (1981) |
| Seattle, WA, USA.;  Starting in Dec. 1980, weekly for 7 weeks | Rain; 28–130 ng g^-1^,  *n* = 5 | Two-step heating, separation of OC in an inert gas at 650 °C | Ogren et al. (1984) |
| Sweden, 12 rural sites;  Apr.–Aug. 1981, monthly | Precipitation;  median 100 (20–600) ng g^-1^, *n* = 60 | Same as above | Ogren et al. (1984) |
| Sierra Blanca, NM (3350 m asl.) and El Paso, TX (1130 m asl.), USA;  winter 1982–1985 | Snowpack;  Sierra Blanca: 4.9 ng g^-1^,  El Paso: 15.9 ng g^-1^ | Thermogram (evolved CO_2_) in air, determined by peak at 450–500 °C with filter transmittance monitoring | Chylec et al. (1987) |
| Hurricane Hill, WA, USA (1500 m asl.); 1983, 1984 | Snowpack; 10.1–18.5 ng g^-1^ | Light absorption (BC): Integrating Plate Method | Clarke and Noon (1985) |
| Cheboygan, MI, USA;  Dec. 1983–Apr. 1984, Dec. 1984–Apr. 1985 | Snow; 72 (28–210) ng g^-1^, *n* = 25 | Thermal separation: heating in an inert gas at 920 °C | Cadle and Dasch (1988) |
| Urban Detroit, MI, USA;  1983–1984 winter 1984– 1985 winter | Precipitation; 130 ng g^-1^, *n* = 15 200 ng g^-1^, *n* = 25 | Same as above, with exception of high loading samples by preliminary thermal separation of OC in air at 350 °C | Dasch and Cadle (1989) |
| Lithuania, 14 rural sites; Dec. 1986–Jun. 1990 | Precipitation; 8–530 ng g^-1^ at Preila site | Light reflectance of filter | Armalis (1999) |
| Mace Head, Ireland; Oct–Nov. 1989 | Rain; 31 (9–94) ng g^-1^,  *n* = 18 | Two-step heating, separation in oxygen at 340 °C | Ducret and Cachier (1992) |
| Azores, Portugal;  Feb. 2003–Jun. 2004 | Rain; 2.8 (0.4–12) ng g^-1^, *n* = 7 | Two-step heating, separation in an inert gas at 600 °C, with charring correction by laser | Cerqueria et al. (2010) |
| Averio, Portugal;  Jan. 2003–Mar. 2004 | Rain;  14 (0.0–65) ng g^-1^, *n* = 33 | Same as above | Cerqueria et al. (2010) |
| Schauinsland, Germany;  Feb. 2003–Aug. 2004 | Rain/snow;  28 (0.0–192) ng g^-1^, *n* = 44 | Same as above | Cerqueria et al. (2010) |
| Sonnblick, Austria (3106 m asl.);  Mar. 2003–Jul. 2004 | Snow;  5.2 (0.0–12) ng g^-1^,  *n* = 23 | Same as above | Cerqueria et al. (2010) |
| K-Puszta, Hungary; Sep. 2002–Jun. 2004 | Rain;  24 (0.0–77) ng g^-1^, *n* = 19 | Same as above | Cerqueria et al. (2010) |
| Three mountain sites in Sierra Nevada, CA, USA;  Feb.–Apr. 2006 | Snow; 5.7, 5.3, 6.9  (1.7–12.9) ng g^-1^ | Thermogram (evolved CO_2_) in O_2_, determined by peak at temperatures of > 480 °C | Hadley et al. (2010) |
| Sapporo and Nemuro, Japan;  Dec. 2007–Mar. 2008 | Snowpack; Sapporo: 0.001–0.8 ng g^-1^, Nemuro:  0.015–0.9 ng g^-1^ | Collected through silver membrane filters, TOR-IMPROVE (Sunset Lab.) | Kuchiki et al. (2009) |
| Sapporo, Japan; Dec. 2007–Mar. 2008,  Dec. 2008–Mar. 2009 | Snowpack; 0.01–0.8 ng g^-1^,  0.005–10 ng g^-1^ | Same as above | Aoki et al. (2011) |
| Northern China,  46 sites; Jan.–Feb. 2010 | Snowpack; (median values) northeast border: 117 ng g^-1^, Inner Mongolia: 340 ng g^-1^, industrial area: 1220 ng g^-1^ | Light absorption (BC): Integrating Sandwich method | Wang et al. (2013) |
| Cape Hedo, Okinawa, Japan: Apr. 1010–Mar. 2013 | Rain; 8.0±4.1–92±76 ng/g | Single-particle soot photometer | Mori et al. (2014) |
| Northwestern China, 44 sites;  Jan.–Feb. 2012 | Snowpack; 5–450 ng g^-1^,  *n* = 284 | Light absorption (BC): Integrating Sandwich method | Pu et al. (2017) |

asl.: above sea level; TOR-IMPROVE: thermo-optical transmittance carbon analysis with Interagency Monitoring for Protected Visual Environments protocol.

**Examples of membrane filters loaded with deposited particles**


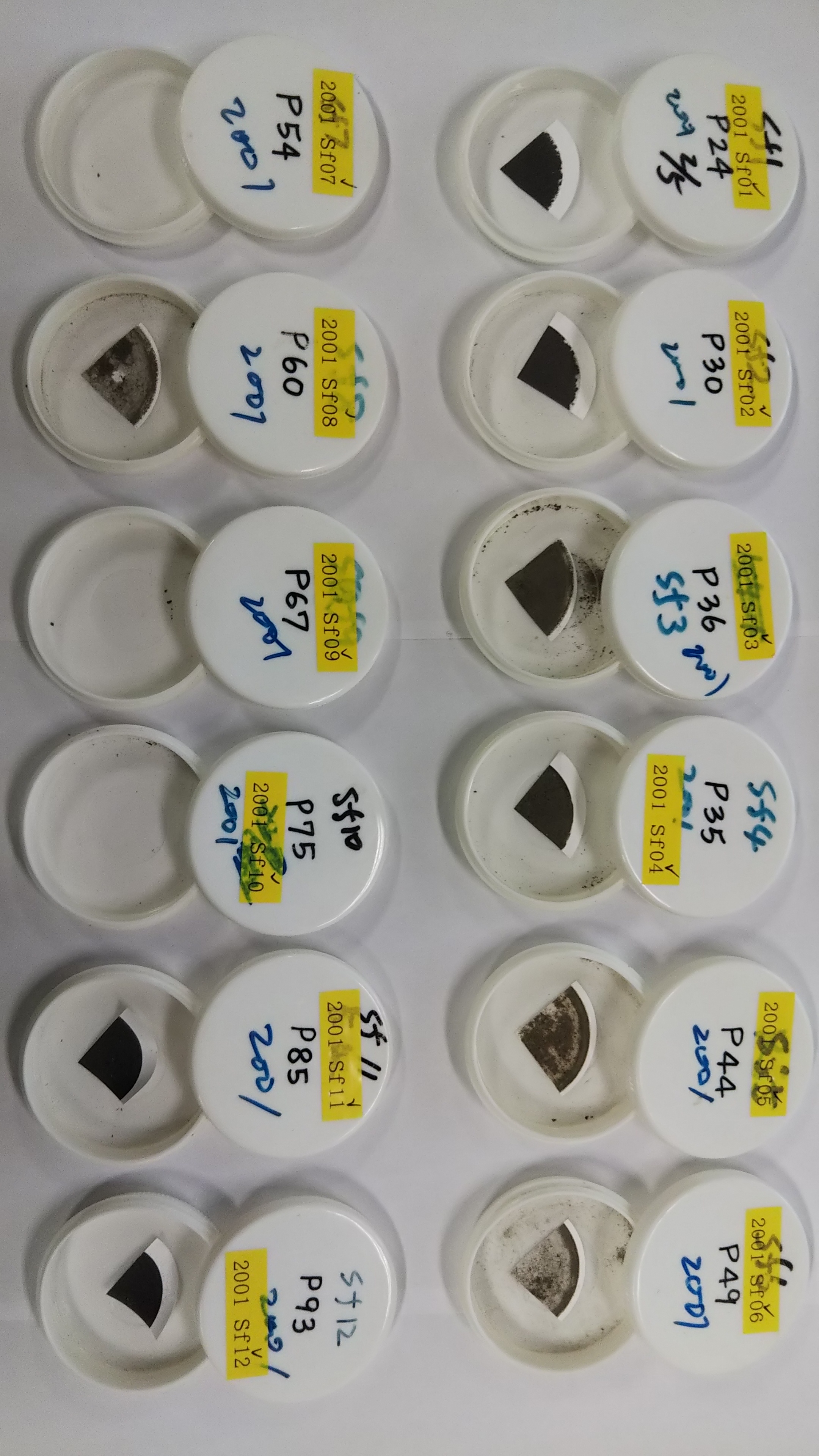


**Figure S3.** Photograph showing examples of membrane filters loaded with deposited particles collected in Sapporo. From upper left to right: samples collected from January to June. From lower left to right: samples collected from July to December 2001. Note that samples from July, September, and October 2001 are lacking because they were totally consumed in the course of EC analysis. EC concentrations in these months were so low that we were required to analyse EC extracted from a large area of membrane filters samples.


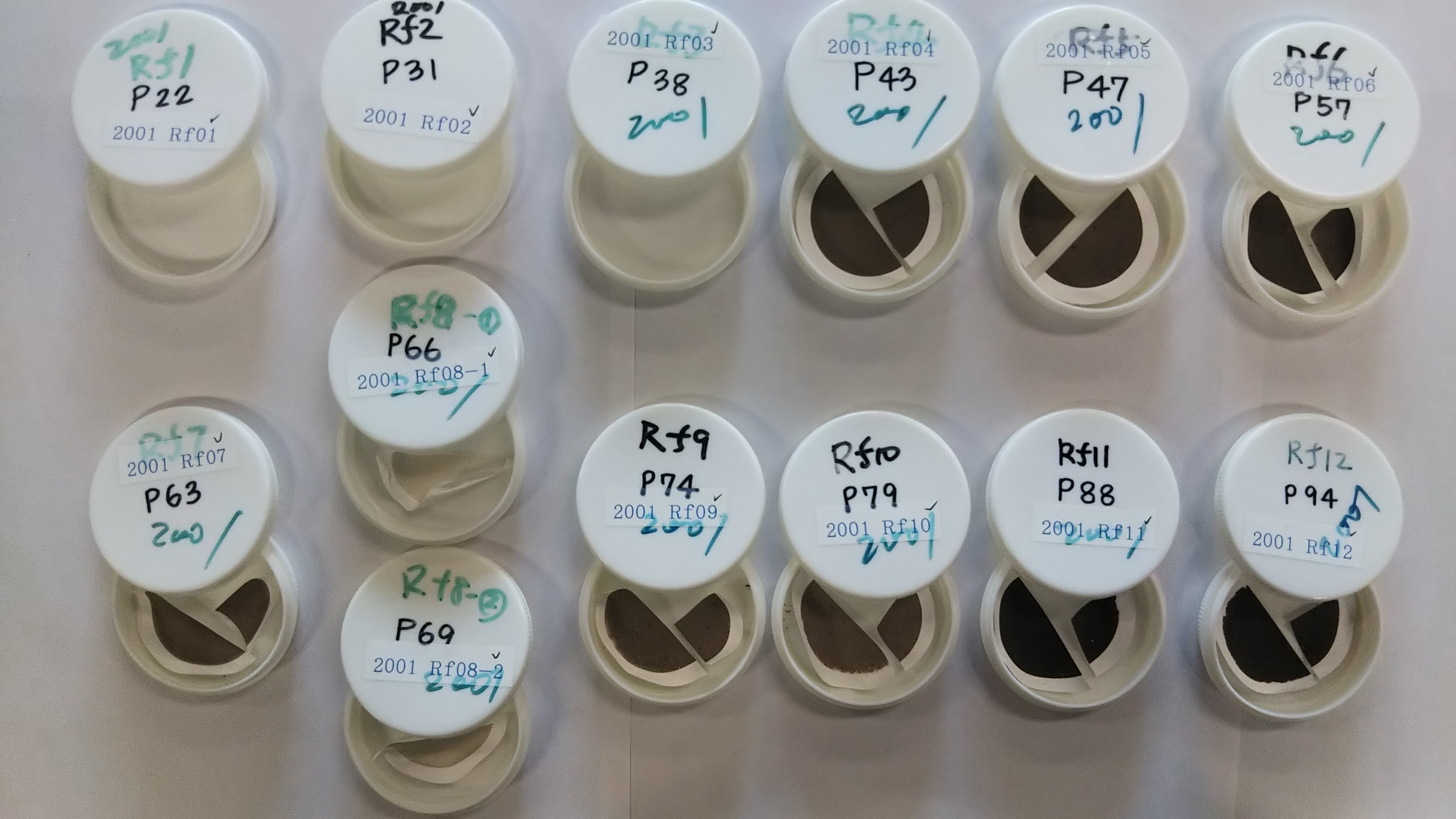


**Figure S4.** Photograph showing examples of membrane filters loaded with deposited particles collected in Rishiri. From upper left to right: samples collected from January to June. From lower left to right: samples collected from July to December 2001. Note that August sample are separated into two filters owing to a large amount of precipitation due to which the collected water in the bottle needed to be collected twice. Samples from January, February, and March 2001 are lacking because they were totally consumed in the course of EC analysis. EC concentrations in these months were so low that we were required to analyse EC extracted from a large area of membrane filters samples.

**References**

Aamaas, B., *et al.* Elemental carbon deposition to Svalbard snow from Norwegian settlements and long-range transport. *Tellus B* **63** (3), 340–351 (2011).

Aoki, T., *et al.* Physically based snow albedo model for calculating broadband albedos and the solar heating profile in snowpack for general circulation models. *J. Geophys. Res. –Atmos*. **116**, D11114 (2011).

Armalis, S. Wet deposition of elemental carbon in Lithuania. *Sci. Total Environ*. **239**, 89–93 (1999).

Cachier, H., Bremond, M. P.& Buat-Menard, P. Determination of atmospheric soot carbon with a simple thermal method. *Tellus B* **41** (3), 379–390 (1989).

Cadle, S. H. & Dasch, J. M. Wintertime concentrations and sinks of atmospheric particulate carbon at a rural location in Northern Michigan. *Atmos. Environ.* **22** (7), 1373–1381 (1988).

Cerqueira, M., *et al*. Particulate carbon in precipitation at European background sites. *J. Aerosol Sci*. **41** (1), 51–61 (2010).

Chylek, P., *et al.* Aerosol and graphitic carbon content of snow. *J. Geophys. Res.–Atmos.* **92** (D8), 9801–9809 (1987).

Clarke, A. D. & Noone, K. J. Soot in the arctic snowpack –a cause for perturbations in radiative-transfer. *Atmos. Environ.* **19** (12), 2045–2053 (1985).

Dasch, J. M. & Cadle, S. H., Atmospheric carbon particulates in the Detroit urban area – Wintertime sources and sinks. *Aerosol Sci. Technol*. **10** (2), 236–248 (1989).

Ducret, J. & Cachier, H. Particulate carbon content in rain at various temperate and tropical locations. *J. Atmos. Chem.* **15** (1), 55–67 (1992).

Grenfell, T. C., Perovich, D. K. & Ogren, J. A. Spectral albedos of an alpine snowpack. *Cold Reg. Sci. Tech.* **4** (2), 121–127 (1981).

Hadley, O. L., Corrigan, C. E., Kirchstetter, T. W., Cliff, S. S. & Ramanathan, V. Measured black carbon deposition on the Sierra Nevada snow pack and implication for snow pack retreat. *Atmos. Chem. Phys.* **10** (15), 7505–7513 (2010).

Jenk, T. M., *et al*. Radiocarbon analysis in an Alpine ice core: record of anthropogenic and biogenic contributions to carbonaceous aerosols in the past (1650­–1940). *Atmos. Chem. Phys.* **6** (12), 5381–5390 (2006).

Kaneyasu, N., Ohta, S. & Murao, N. Seasonal variation in the chemical composition of atmospheric aerosols and gaseous species in Sapporo, Japan, *Atmos. Environ.* **29**, 1559–1568 (1995).

Kuchiki, K., Aoki, T., Tanikawa, T. & Kodama, Y. Retrieval of snow physical parameters using a ground-based spectral radiometer. *Appl. Opt*. **48** (29), 5567–5582 (2009).

Lavanchy, V. M. H., Gaggeler, H. W., Schotterer, U., Schwikowski, M. & Baltensperger, U. Historical record of carbonaceous particle concentrations from a European high-alpine glacier (Colle Gnifetti, Switzerland). *J. Geophys. Res. –Atmos.* **104** (D17), 21227–21236 (1999).

Liu, X., Xu, B., Yao, T., Wang, N. & Wu, G. Carbonaceous particles in Muztagh Ata ice core, West Kunlun Mountains, China. *Chin. Sci. Bull.* **53** (21), 3379–3386 (2008).

Ming, J., *et al*. Black carbon record based on a shallow Himalayan ice core and its climatic implications. *Atmos. Chem. Phys.* **8** (5), 1343–1352 (2008).

Mori, T. *et al*. Wet deposition of black carbon at a remote site in the East China Sea. *J. Geophys. Res. –Atmos.* **119**, 10485–10498 (2014).

Ogren, J. A., Charlson, R. J. & Groblicki, P. J. Determination of elemental carbon in rainwater. *Anal. Chem.* **55** (9), 1569–1572 (1983).

Ogren, J. A., Groblicki, P. J. & Charlson, R. J. Measurement of the removal rate of elemental carbon from the atmosphere. *Sci. Total Environ*. **36**, 329–338 (1984).

Pu, W., *et al*. Properties of black carbon and other insoluble light-absorbing particles in seasonal snow of northwestern China. *Cryosphere* **11** (3), 1213–1233 (2017).

Seinfeld, J. H. & Pandis, S. N. Atmospheric Chemistry and Physics: From air pollution to climate change 444 (John Wiley&Sons, 1998).

Thevenon, F., Anselmetti, F. S., Bernasconi, S. M. & Schwikowski, M., Mineral dust and elemental black carbon records from an Alpine ice core (Colle Gnifetti glacier) over the last millennium. *J. Geophys. Res. –Atmos*. **114**, D17102 (2009).

Torres, A., Bond, T. C., Lehmann, C. M. B., Subramanian, R. & Hadley, O. L. Measuring organic carbon and black carbon in rainwater: Evaluation of methods. *Aerosol Sci. Technol.* **48** (3), 239–250 (2014).

Wang, X., Doherty, S. J. & Huang, J. Black carbon and other light-absorbing impurities in snow across Northern China. *J. Geophys. Res. –Atmos.* **118**, 1471–1492 (2013).

Xu, B. Q., *et al.* Deposition of anthropogenic aerosols in a southeastern Tibetan glacier. *J. Geophys. Res. –Atmos.* **114**, D17209 (2009).
